# Recent developments in computer assisted rehabilitation environments

**DOI:** 10.1186/2054-9369-1-22

**Published:** 2014-10-20

**Authors:** Rob van der Meer

**Affiliations:** MeerHealth, Riënzistraat 41, 2555 JT ‘s-Gravenhage, The Netherlands

**Keywords:** Computer assisted rehabilitation environment, Virtual reality, Trauma, Amputations, Rehabilitation, Post-traumatic stress disorder, Military medicine

## Abstract

Computer Assisted Rehabilitation Environment (CAREN) is a system that integrates a training platform (motion base), a virtual environment, a sensor system (motion capture) and D-flow software. It is useful for both diagnostic and therapeutic use. The human gait pattern can be impaired due to disease, trauma or natural decline. Gait analysis is a useful tool to identify impaired gait patterns. Traditional gait analysis is a very time consuming process and therefore only used in exceptional cases. With new systems a quick and extensive analysis is possible and provides useful tools for therapeutic purposes. The range of systems will be described in this paper, highlighting both their diagnostic use and the therapeutic possibilities. Because wounded warriors often have an impaired gait due to amputations or other extremity trauma, these systems are very useful for military rehabilitative efforts. Additionally, the virtual reality environment creates a very challenging situation for the patient, enhancing their rehabilitation experience. For that reason several Armed Forces have these systems already in use. The most recent experiences will be discussed; including new developments both in the extension of the range of systems and the improvement and adaptation of the software. A new and promising development, the use of CAREN in a special application for patients with post-traumatic stress disorder (PTSD), will also be reviewed.

## Introduction

The use of Improvised Explosive Devices (IEDs) in recent conflict often results in complex orthopedic and neurological trauma, which may include limb loss, spinal cord injuries and traumatic brain injury. Survival rates have significantly increased with mortality among US troops at 8.5% in World War I, 3.3% in World War II, 2.6% in The Vietnam War. In recent operations, mortality has increased to 4.8%, most likely a result of a decreased number of Killed in Action (20.0% in WW II as compared to 13.8% in recent operations) [1]. Unfortunately, many warriors sustain limb amputations, as many as 3.5%-14.0% trauma admissions in recent operations [2]. The primary rehabilitation goal for these individuals is to provide them with an expedited recovery and progressive reintroduction into the civilian or active duty populations. In this relatively young population, new challenging rehabilitation methods are ideal. The Computer Assisted Rehabilitation Environment (CAREN) is such a system [3].

### Virtual reality

Virtual reality in medicine, and more specifically in rehabilitation medicine, is a developing area. Lohse et al. [4] carried out a literature study comparing Virtual Reality therapies (VR) to conventional therapies (CT) and commercially available gaming systems (CG) in stroke patients. There was a wide range of CTs, like treadmill gait training, balance training, postural control, creative therapy, etc. The study showed that virtual reality moderately improved outcomes compared to conventional therapy in adults post-stroke. Current CG interventions have been too few and too small to assess potential benefits of CG. Future research in this area should aim to clearly define conventional therapy, report on participation measures, consider motivational components of therapy, and investigate commercially available systems in larger randomized clinical trials (RCTs). McEwen et al. [5] showed that virtual reality exercises in stroke patients improved mobility-related outcomes. Another study addressed upper extremity dysfunction after stroke. The traditional rehabilitation approaches based on one-to-one physiotherapist-patient interaction are evidence based. However, recent evidence is enlightening the possibility that innovative approaches, based on the augmentation of specific kinematic feedbacks, could enrich the rehabilitation environment, possibly leading to a significant improvement of the motor function. This enrichment could potentially facilitate the physiological activation of the brain areas devoted to motor relearning. As a consequence, reinforced feedback in virtual environment can promote the recovery of motor function in post-stroke patients, by means of regular, intensive, and supervised training [6]. The literature available supports the conclusion that virtual reality therapies have an added value in motor learning and stability training [7–9].

### CAREN

**Figure 1:**
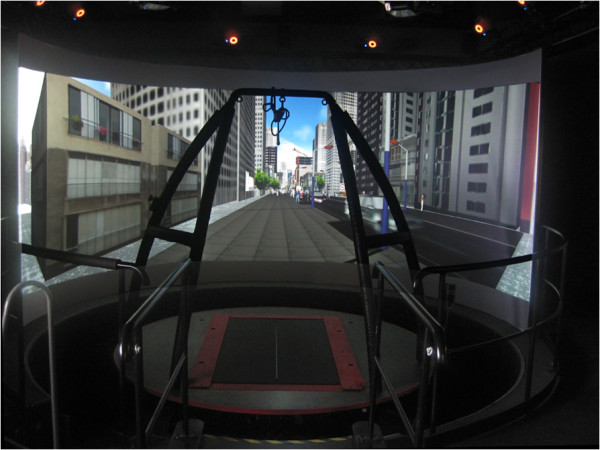
**CAREN platform with a 180° screen.**

**Figure 2:**
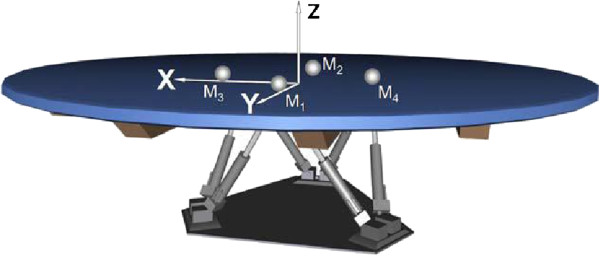
**Six degrees of freedom (DOF) platform.**

**Figure 3:**
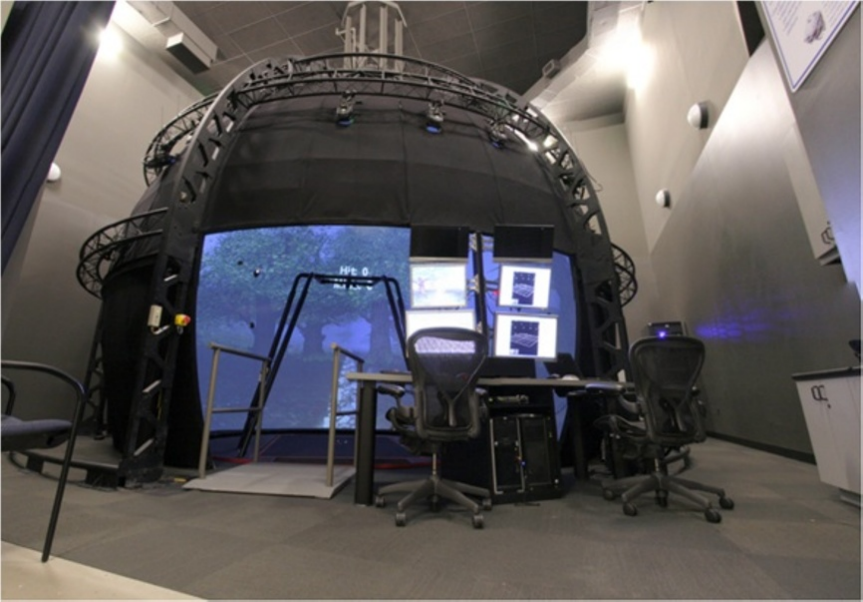
**CAREN in a dome configuration.**

The CAREN system consists of a motion capture system and a base platform, driven by hydraulic and mechanical actuators (Figure 1). The base where the user stands is designed with force plates and a treadmill, with up to 6 degrees of freedom (Figure 2). This allows the operator to generate visual and physical perturbations that require the user to make dynamic responses during their gait patterns. The CAREN system may also be equipped with varying degrees of virtual reality immersion ranging from a flat video, dual-channel audio, and theater in its “base” model to a 360° surround sound dome enclosure in its “high end” version (Figure 3). Real-time motion tracking technology enables the CAREN system to track patient movements. The CAREN system is equipped with a safety harness for the patient. The whole system is controlled by the “D-flow software”, which can be specifically adapted and customized to the user specifications. Depending on the model, CAREN is spacious equipment that requires the adequate housing infrastructure.

CAREN operates based on biofeedback principles. In a recent publication, Giggins et al. [10] describe the different types of biofeedback in rehabilitation. Biofeedback has been widely used in rehabilitation for several decades. It is the technique of providing biological information to patients that would otherwise be unknown. Biofeedback can be divided into biomechanical and physiological biofeedback. Examples of physiological feedback are neuromuscular (Electromyography, EMG), cardiovascular (heart rate, blood pressure) and respiratory. Biomechanical biofeedback involves measurements of the movement, postural control and forces produced by the body. Inertial sensors, force plates, electrogoniometers, pressure biofeedback units and camera based systems are all measurement devices which can be used to provide biomechanical biofeedback. Biomechanical biofeedback is more complex than physiological biofeedback, as one measurement device can be used to deliver different types of biomechanical feedback. For example, a force plate can be used to deliver both feedback on force and postural control. Several of these biofeedback modalities are all integrated in the CAREN system.

### Literature review

CAREN was developed in the 1990’s and with a limited use in the beginning. This is reflected by the literature that is rather limited and mainly focused on more technical aspects of the tool and oriented on the clinical use for stroke patients only. Specific reports on its use in the military and with amputees are scarce. Here we review a selection of current literatures.

Van der Eerden et al. [11] were one of the first to describe CAREN. They described CAREN as made by customizing hardware and developing software, to enable detailed measurements of motion of a subject in response to perturbations from the computer driven motion platform. After feeding the data into a human body model simulation, joint moments of force and muscle activation can be calculated. From the time patterns of these responses, inferences can be made concerning the motor programs the subjects launch. Any primary problem in a motor program, resulting in functional failure or inadequacy, can be identified down to the joint and muscle group. Secondary adaptations of patients to a limitation in the periphery (such as lack of muscle force) can be separated from the primary ones. Especially the understanding of compensation strategies in patients may lead to a better therapeutic attitude. The authors conclude that CAREN offers not only a test environment, capable of almost unlimited exploratory behaviors for patients, but also an outstanding tool for motor control research.

Barton et al. [12] states that the functionality of the usual movable platforms used in human balance studies is limited, as the rotations are allowed around a pre-defined axes, which typically runs close to the platform's surface and therefore cannot be used to directly investigate control mechanisms of proximal joints. They determined the CAREN platform’s axes of rotation in all three planes and found the CAREN platform’s consistency to be comparable to validated methods for determining joint axes. They conclude that the CAREN platform is a valuable tool to generate perturbations by rotating the supporting surface around a kinematically reconstructed joint axes. The described method could serve as a benchmark for other CAREN systems. The accurate translation of the CAREN platform’s axes of rotation into arbitrary locations overcomes a major limitation of other moving platforms. The benefit of rotating the supporting platform around a joint’s axis rather than around an axis outside the body is that the joint is purely rotated without being translated thus removing the confounding effects of translation on balance. The ability to apply isolated rotational perturbations on targeted joints while standing, opens up the possibility to focus on the role of individual joints in balance mechanisms.

Maksoud et al. [13] also studied virtual reality technology and states that “it offers the opportunity to expose patients to complex physical environments, without physical danger and thus provides a wide range of opportunities for locomotor training, or the study of human postural and walking behavior”. A clinical study has shown that post-stroke patients are able to adapt and benefit from this system, wherein they walk into virtual environments on a self-paced treadmill mounted on a platform with 6 degrees of freedom. This platform is programmed to mimic changes in the terrain encountered in the virtual environments. While engaging in these virtual environments, excessive trunk movements and speed alterations have been observed, especially during the pitch perturbations accompanying uphill or downhill terrain changes. The aim of this study was to determine an optimal solution to simulate walking in real life when engaging in virtual environments.

Hawkins et al. [14] studied the relationship between performance and difficulty by altering game velocity and surface perturbations in a virtual game environment. Performance deteriorates as game difficulty increases, when changing game velocity and surface perturbations. Adjustment of both game velocity and the introduction of surface perturbations independently appear to be simple and effective methods of customizing task difficulty as a function of patients’ motor ability during rehabilitation.

**Figure 4:**
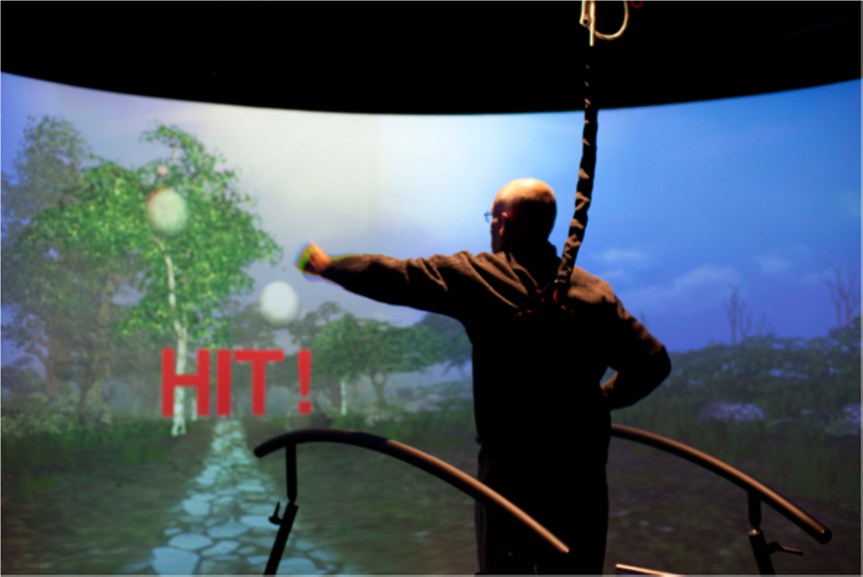
**Dual tasking which means the subject on the CAREN is offered a scenario to walk through and at the same time performs certain other tasks like catching balls.**

Kizony et al. [15] studied dual-tasking, specifically in people with neurological deficits (Figure 4). The authors designed a study to test the feasibility of using a virtual functional environment for the examination of dual-tasking and to determine the effects of dual-tasking on gait parameters in people with stroke and age-matched healthy controls. Participants walked on a self-paced treadmill while viewing a virtual grocery aisle projected onto a screen placed in front of them. They were asked to walk through the aisle (single task) or to walk and select (“shop for”) items according to instructions delivered before or during walking (dual-tasking) (Figure 4). Overall, the stroke group walked slower than the control group in both conditions, whereas both groups walked faster over ground than on the treadmill. The stroke group also showed larger variability in gait speed and shorter stride length than the control group. There was a general tendency to increase gait speed and stride length during dual-task conditions. However, a significant effect of dual-tasking was found only in one dual-task condition for gait speed and stride duration variability. All participants were able to complete the task with minimal mistakes. They conclude that it is feasible to use a functional virtual environment for investigation of dual-tasking. Different gait strategies, including an increase or decrease in gait speed can be used to cope with the increase in cognitive demands required for dual-tasking.

Isaacson et al. [3] described how CAREN may improve physical and cognitive rehabilitation for wounded warfighters. They employed the CAREN system to assist service members who had sustained IED injuries during Operation Enduring Freedom, Operation Iraqi Freedom, and Operation New Dawn. They describe that the complex nature of warfighter injuries, present unique rehabilitation challenges that demand new tools for quick return to active duty or the civilian community. They describe the CAREN system as unique, in that it allows the wounded warriors to be immersed in a realistic clinical environment, while therapist and physicians collect kinematic and kinetic data in order to plan future rehabilitation regimens. In everyday life, warfighters with lower extremity trauma may experience uneven terrain, cracks in pavements, slippery conditions, etc. All potential scenarios may increase fall risk or injury. However, when using the CAREN system, specific physical perturbations may simulate these environmental conditions in a safer and controlled setting. New rehabilitation methods and gait/prosthetic limb training may be developed for these individuals to mitigate falling risks outside of the clinic. They conclude that the CAREN system is a dynamic rehabilitation aid and may be a translational tool for collecting biomechanical and physiological data during prosthetic training. As such, rehabilitation regimens may be patient specific.

### Experience

The Netherlands Armed Forces have a Military Rehabilitation Center, where all military rehabilitation patients are being treated. Almost 40% of the population is civilian patients. In Table 1, you see the distribution of the patient categories. As expected, the great majority of stroke patients (90%) were civilians, whereas 80% of the amputation patients were military.

**Table 1:** **Distribution of patient categories**

Diseases	Rate (%)
Cerebrovascular accidents	20
Anterior cruciate ligament injury	15
Amputation	15
Multi-trauma	10
Vertigo	10
Miscellaneous	30

In a study at the Dutch Central Military Rehabilitation Center, 50 subjects underwent training on the CAREN [16]. They were asked to give their opinion on eight questions, using a Visual Analog Score by placing a mark on a continuous 10 cm long line. The general domains addressed were the following: Relationship with the therapist, goal oriented therapy, the approach of the therapists, and the way the patient thinks about the general therapeutic contact. Specifically for CAREN the following was investigated: The attributed importance of training on the CAREN-system to the rehabilitation process, whether the rehabilitation process accelerated by the CAREN-system, whether the amount of the therapy is considered challenging and will the training transfer to the real world. All domains scored above 8, which is a satisfactory level. Interestingly, patients point out that they think that training on the CAREN system accelerates the rehabilitation process, has an important role in their recovery, and is challenging, and the transfer of training to the real world is noticeable.

### Post-Traumatic Stress Disorder (PTSD)

In a recent paper by Vermetten et al. [17] a report is given on developments of the use of the CAREN system for mental health problems in the military. They describe that although the symptoms of PTSD in the general and military population seem very similar, combat-related PTSD is typically thought to be more severe due to the repeated and prolonged exposure of traumatic events. Therapeutic adherence is a problem in military population that compromises treatment efficacy. Therefore, a new potential supplementary treatment was specially designed for patients with combat related-PTSD. This intervention is called Military Motion Memory Desensitization and Reprocessing (3MDR). The treatment incorporates key elements of successful treatments as Virtual Reality Exposure and Eye Movement Desensitization Reprocessing, and adds motion to the condition. The authors aimed at designing a treatment procedure that preserved dual-task processing principle, but also introduced new engagement by performing the desensitization during motion by walking on a treadmill. Moreover, they aimed at exposure to real high-affect pictures of a deployment setting. These pictures are selected by the patients and must have a link to the traumatizing event or environment. Subjects walk a repetitive cycle while viewing the high-affect pictures of deployment scenes. Dual-task processing was maintained by an oscillating ball. Aspects of presence are adhered to in order to maximize possible positive outcome. The authors describe the therapy in 2 patients and conclude that the results of the two cases suggest that the 3MDR treatment is a successful treatment that goes further into the patients affect, whereas traditional treatments may fail to lead to further results. The presence was highly appreciated. They plead for further research with more patients to be performed to obtain more reliable results.

### Technical developments

**Figure 5:**
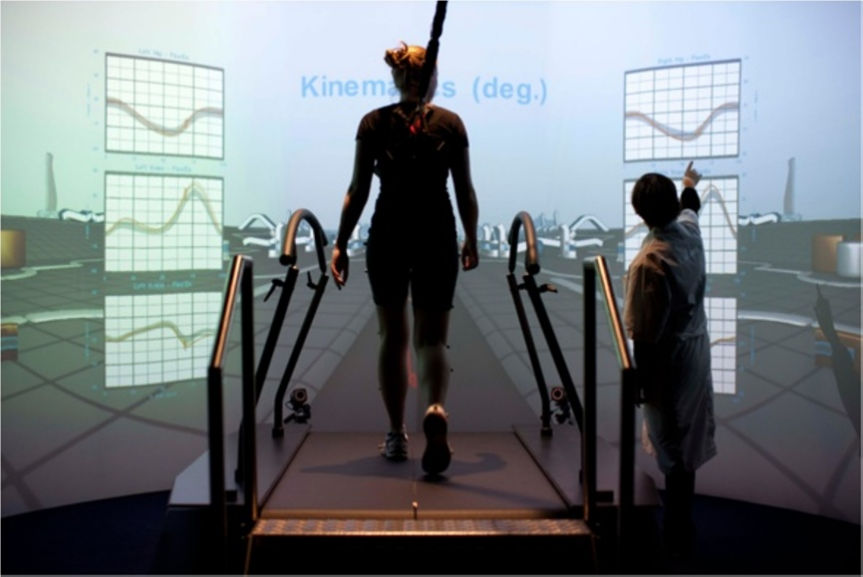
**Real time gait analysis (the subject is walking on a treadmill and the gait analysis tools are projected on line on the screen, the therapist can interpret the results and adapt the performance of the patient instantaneously).**

**Figure 6:**
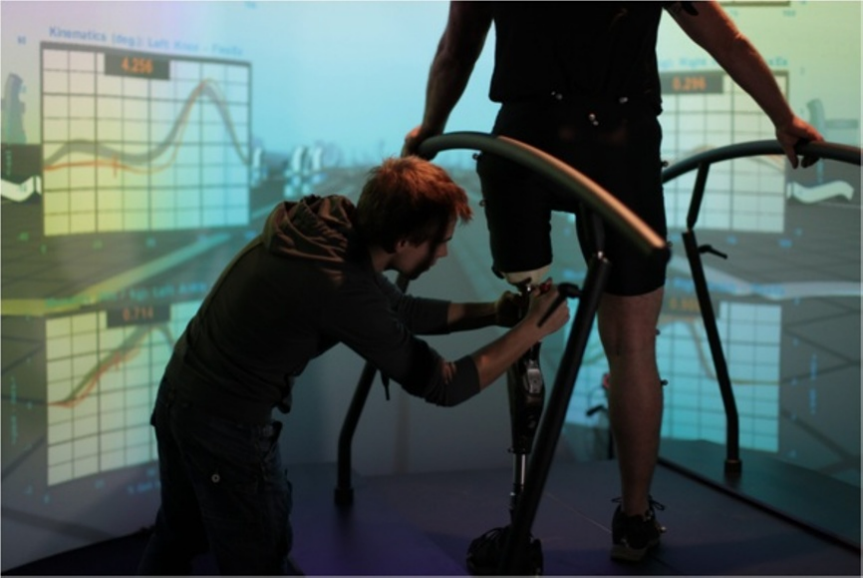
**GRAIL, a treadmill, combined with a screen on which a scenario is projected and performance of the patient is required.**

The CAREN system is mainly designed for a Research and Development environment. It has certain infrastructural requirements, needs a significant investment and a higher level of technical development. Therefore, MOTEK Medical (Amsterdam, The Netherlands) developed other similar systems for clinical use [18].The GRAIL, Gait Real-Time Analysis Interactive Laboratory (Figures 5 and 6), is a tool for use in regular rehabilitation medicine as a gait lab. Instead of the 6DOF platform, GRAIL uses a dual belt treadmill. GRAIL needs less space and provides quick output of results. It offers a total package solution for clinical gait analysis and gait training using the latest technologies. Besides the functionality of a traditional gait lab, GRAIL provides real-time gait analysis, gait off-line analysis tool (GOAT: a gait report directly after the session that facilitates video analysis and post-processing/filtering options for data analysis). Recording multiple steps makes it possible to calculate averages, standard deviations and variations of gait parameters over time, functional gait analysis for the identification of pathologic responses to perturbations, visual challenges, fast pitch, and sway and belt speed changes, enable identification and quantification of compensatory strategies or dynamic stability and gait training. The design makes the wide spread use in rehabilitation feasible.

### STABLE

**Figure 7:**
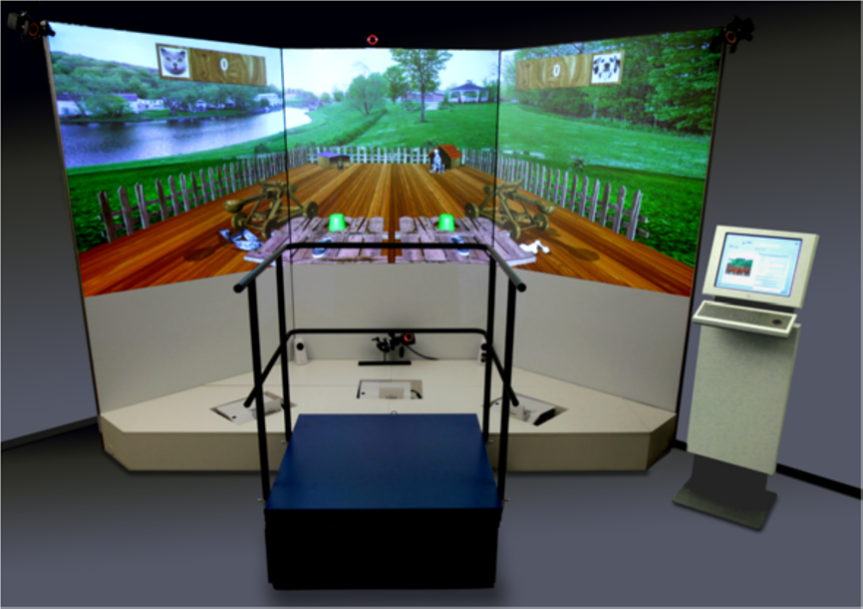
**STABLE, a platform with diagnostic facilities and training programmes, mainly focused at stability training.**

Stability and Balance Learning Environment (Figure 7) is a system for the assessment and training of postural stability and balance-related disorders in neurological petients, orthopedic patients, patients with musculo-skeletal complaints, and especially in the elderly with increased risk of falling. STABLE uses a force plate and motion capture, and a projection screen. One can obtain objective and quantitative outcome measures for stability and balance alongside fun and motivating exercise games to train stability and balance with reduced risk of falling.

## Conclusion

CAREN is a useful tool for rehabilitation in the military. Although the literature is limited and research is still in progress, the first results are promising in terms of both clinical results and patient acceptance. In The Netherlands Armed Forces as well as in other nations, it is a valuable tool in the rehabilitation programs for wounded warriors. New development in the use of CAREN for the treatment of-combat related PTSD is rather positive. Technical developments will make the use of Virtual Environments in rehabilitation medicine more widespread and feasible.

### Consent

Written informed consent was obtained from the patient for the publication of this report and any accompanying images.

## Author details

Prof. Rob van der Meer, MD, was Brigadier General (retired), Surgeon General of The Netherlands (retired), Chairman COMEDS of NATO (retired), Chairman Pan-European Working Group ICMM(retired), and his active position is the CEO of MeerHealth and the Consultant with MOTEK Medical bv, Riënzistraat 41, 2555 JT ‘s-Gravenhage, The Netherlands.

## Abbreviations

CAREN: Computer assisted rehabilitation environment
CG: Commercially available gaming systems
CT: Conventional therapies
DOF: Degrees of freedom
EMG: Electromyography
GRAIL: Gait real-time analysis interactive laboratory
IEDs: Improvised explosive devices
3MDR: Military motion memory desensitization and reprocessing
PTSD: Post-traumatic stress disorder
STABLE: Stability and balance learning environment
VR: Virtual reality.
